# Combined Intravitreal Anti-VEGF and Photodynamic Therapy versus Photodynamic Monotherapy for Polypoidal Choroidal Vasculopathy: A Systematic Review and Meta-Analysis of Comparative Studies

**DOI:** 10.1371/journal.pone.0110667

**Published:** 2014-10-24

**Authors:** Wei Wang, Miao He, Xiulan Zhang

**Affiliations:** Zhongshan Ophthalmic Center, State Key Laboratory of Ophthalmology, Sun Yat-Sen University, Guangzhou, People's Republic of China; MGH, MMS, United States of America

## Abstract

**Purpose:**

The aim of this study was to evaluate the efficacy and safety of photodynamic therapy (PDT) combined with intravitreal vascular endothelial growth factor (VEGF) inhibitors compared to those of PDT alone in the treatment of polypoidal choroidal vasculopathy (PCV).

**Methods:**

A systematic search of Pubmed, Embase, and the Cochrane Library was performed to identify all comparative studies that compared the outcomes of the two approaches. Outcomes of interest included visual outcomes, anatomic variables, and adverse events.

**Results:**

Two randomised controlled trials and nine retrospective studies including a total of 543 cases were identified. At three and six months post-injection, no significant difference in visual acuity was found in the combined therapy group compared with the PDT monotherapy group, with pooled weighted mean differences (WMDs) of 0.074 (−0.021, 0.17) at three months and 0.082 (−0.013, 0.18) at six months. However, the mean changes in visual acuity at month 12 in the combined therapy group were significantly better than those in the PDT monotherapy group, with pooled WMDs of 0.11 (0.012, 0.21). Similar efficacy was found at 24 months (WMD: 0.21; 95%CI: 0.054, 0.36; P = 0.008). Patients in the combined therapy group also might benefit from reduced retinal haemorrhage (OR: 0.32; 95% CI: 0.14, 0.74; P = 0.008). Polyp regression, recurrence of PCV, central retinal thickness reduction, and pigment epithelial detachment resolution did not differ significantly between the two treatments.

**Conclusions:**

Combined treatment appeared to result in better visual acuity and lower retinal haemorrhage. However, combined treatment did not affect the resolution and recurrence of lesions. Given the inherent limitations of the included studies, future well-designed RCTs are awaited to confirm and update the findings of this analysis.

## Introduction

Polypoidal choroidal vasculopathy (PCV) is a sight-threatening disease, which is relatively prevalent in Asian populations [1]. About half of the eyes that did not undergo treatment had persistent leakage or repeated bleeding with vision loss. Pathogenesis of PCV is not fully understood, but vascular endothelial growth factor (VEGF) may have a role in pathogenesis. VEGF concentrations in the aqueous were found to be markedly increased in PCV eyes compared to controls. Treatment strategies for PCV include thermal laser photocoagulation, verteporfin photodynamic therapy (PDT), anti- VEGF therapies, and the combination therapy of PDT with anti-VEGF. However, there is still no consensus regarding the most effective treatment for PCV [2], [3]. Currently, PDT is widely used in the treatment of PCV, as various studies have demonstrated that PDT can result in visual improvement [4]–[7]. However, haemorrhagic complications after PDT have been reported in up to 30% of eyes, and repeated PDT results in significant choroidal hypoperfusion [5], [7]–[9]. With the introduction of anti-VEGF drugs in ophthalmology community, intravitreal anti-VEGF agents were widely used for neovascular disease such as wet age related macular degeneration and PCV. Unlike for age related macular degeneration, anti-VEGF compounds by themselves do not work well in PCV. Thus, combination therapy comprising PDT and anti-VEGF drugs, such as bevacizumab and ranibizumab, become another treatment choice for PCV. Because increased expression of VEGF has been found in PCV patients following PDT, the combined therapy has been thought to result in additional or complementary effects [2].

To date, several studies comparing PDT combined with anti-VEGF drugs and PDT monotherapy have been conducted [8], [10]–[19]. However, most are small series with conflicting results, and no definitive conclusions regarding objective differences in outcomes have been reached. For example, Gomi [19] and colleagues reported significantly better results with PDT plus anti-VEGF therapy compared with PDT monotherapy one year after treatment. However, according to the study of Rouvas [8] and colleagues, PDT resulted in a significantly better outcome than PDT with ranibizumab after one year of followup. Therefore, we performed a systematic review and meta-analysis of the available published literature to compare the outcomes of the two approaches.

## Methods

This study was reported in accordance with the Preferred Reporting Items for Systematic Reviews and Meta-Analyses (PRISMA) statement (Checklist S1) [20]. All stages of study selection, data extraction, and quality assessment were performed independently by two reviewers (W.W. and M.H). Any disagreement was resolved via discussion and consensus.

### 1. Literature search

A systematic search of Pubmed, Embase, and the Cochrane Library was performed to identify relevant studies up to September 2013. No time or language restrictions were applied. The following terms, adapted for each database, were used for the searches: (“polypoidal choroidal vasculopathy” OR PCV) AND (“angiogenesis inhibitors” OR “endothelial growth factors” OR VEGF OR lucentis OR ranibizumab OR bevacizumab OR avastin) AND (“photodynamic therapy” OR PDT).The Related Articles function was also used to broaden the search, and the computer search was supplemented with manual searches of the reference lists of all retrieved studies, review articles, and conference abstracts.

### 2. Inclusion and exclusion criteria

All available randomised controlled trials (RCTs) and non-randomized comparative studies (cohort or case–control studies) that compared combined PDT with anti-VEGF therapy with PDT monotherapy in all age groups, and that had at least one of the quantitative outcomes mentioned in the next section of this paper, were included. Editorials, letters to the editor, review articles, case reports, meeting abstracts, and animal experimental studies were excluded. The studies on PDT combined with anti-inflammatory compounds and triple therapy were also excluded.

### 3. Data extraction

The data were extracted independently by two reviewers (W.W. and M.H.). Disagreements were resolved through discussion. The following information was extracted from each study: first author; year of publication; study design; inclusion and exclusion criteria; number of patients in each group; characteristics of the study population; and outcomes of interest. The numbers of withdrawals and patients reporting adverse events were also recorded. For publications reporting on the same study population, the article reporting the results of the last end point was included, and data that could not be obtained from this publication were obtained from the others.

### 4. Outcome measures

The following outcomes were used to compare combined therapy and PDT alone. (1) Visual outcomes: mean visual acuity (VA) change at months 3, 6, 12, and 24; and proportion of eyes with improved, stable, and deteriorated vision after each treatment at months 12 and 24. After assessing VA at each follow-up visit, the patients were categorised into three groups based on their VA change from baseline: improved, stable, and deteriorated VA. (2) Anatomical outcomes: mean change in central retinal thickness (CRT) at months 6; regression rates of polyps at months 3 and 12; resolution of pigment epithelial detachment (PED) at 12-month followup; and recurrence rate of PCV. (3) Adverse events: incidence of retinal haemorrhage.

### 5. Quality assessment

The methodological quality of RCTs was assessed using the Cochrane Risk of Bias Tool [21]. The methodological quality of observational studies was assessed using the modified Newcastle–Ottawa scale [22], which consists of three factors: patient selection, comparability of the study groups, and assessment of outcome. A score of 0–9 (allocated as stars) was allocated to each study except RCTs. RCTs and observational studies achieving seven or more stars were considered to be of high quality.

### 6. Statistical analysis

Weighted mean differences (WMDs) and odds ratios (ORs) were used to compare continuous and dichotomous variables, respectively. All outcomes were reported with 95% confidence intervals (CIs). Considering the different clinical characteristics among study groups and the variation in sample sizes, we assumed that heterogeneity was present even when no statistical significance was identified; therefore, we decided to combine data by using a random effects model to achieve more conservative estimates. The change in VA of each eligible arm of the individual studies was calculated as the difference between the value at baseline and those at the different follow-up times, and the variance was computed as the weighted mean of their variances. The WMD was then computed as the between-treatment difference in the visual changes from baseline.

Statistical heterogeneity among studies was assessed using the chi-squared test with significance set at P<0.10. The percentage of heterogeneity was evaluated using the I^2^ statistic, which ranges from 0% to 100%, with 0% representing no heterogeneity and larger values representing greater heterogeneity (I^2^ = 0–25% indicates no or mild heterogeneity; I^2^ = 25–50% indicates moderate heterogeneity; I^2^ = 50–75% indicates large heterogeneity; and I^2^ = 75–100% indicates extreme heterogeneity) [23].

Subgroup analysis was performed according to type of anti-VEGF agents, frequency of anti-VEGF agent, patient history of previous treatment, and intevals between PDT and anti-VEGF therapy. Sensitivity analysis was performed by excluding each study, one by one, and recalculating the combined estimates based on the remaining studies. Only outcomes of interest that were reported in more than three studies were included in the sensitivity analysis. Potential publication bias was evaluated by Begg's and Egger's tests. All analyses were performed with Stata Version 12.0 (StataCorp, College Station, TX). A p value<0.05 was considered significant, except where otherwise specified.

## Results

### 1. Characteristics of eligible studies

**Figure 1:**
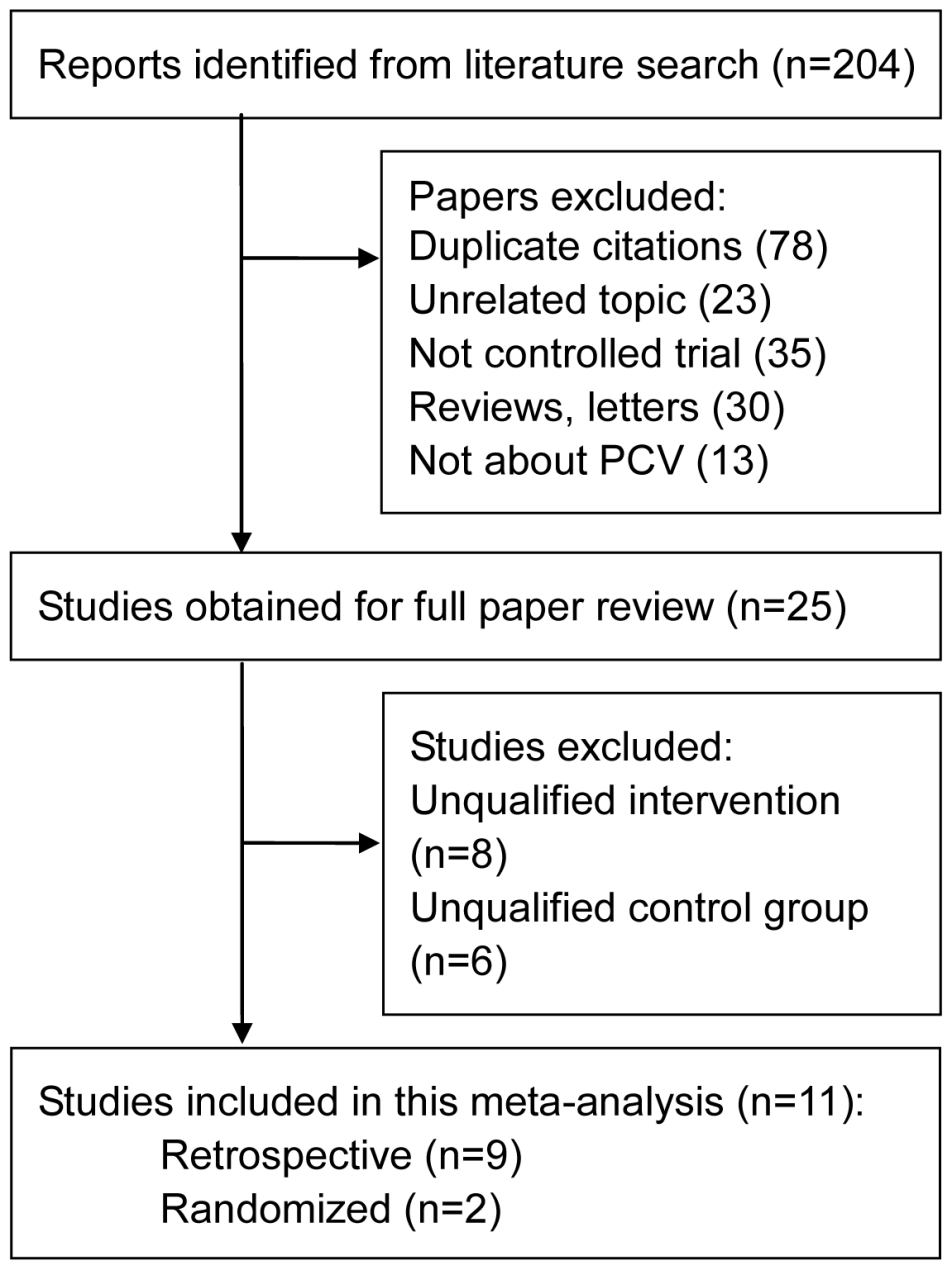
Flow diagram of included studies for this meta-analysis.

Eleven studies [8], [10]–[19] including 543 cases (253 cases of combined PDT with anti-VEGF therapy and 290 cases of PDT monotherapy) fulfilled the predefined inclusion criteria and were included in the final analysis (Figure 1). The characteristics of the included studies are shown in Tables 1 and 2. Among the included studies, there were two small-sample RCTs and nine retrospective comparative studies.PCV was confirmed by ICGA in all studies. The inclusion criteria of the patients in these studies were summarized in table S1. The ICGA and OCT were used in the same way in all subcomponents of these studies. All of the retrospective comparative studies scored seven stars or higher and were considered to be of high quality.

**Table 1:** Characteristics of studies included in the meta-analysis.

Trial	Design	Centre	Location	Follow-up	No. of eyes		Mean Age(year)		Sex (M/F)	
					Combine	PDT	Combine	PDT	Combine	PDT
Lee (2013)	RCT	1	Korea	3 m	12	8	63.68±8.78	66.33±7.85	11/1	5/3
Saito (2013)	Retro	1	Japan	≥24 m	25	32	74.0±8.6	75.0±6.5	17/8	27/5
Lee (2012)	Retro	1	Taiwan	≥24 m	36	33	67.8±9.8	65.6±9.5	21/15	18/15
Koh (2012)	RCT	7	Hong Kong, Singapore, South Korea, Taiwan, Thailand	6 m	19	21	63.8±8.30	62.2±9.77	11/8	15/16
Kim (2011)	Retro	1	Korea	>12 m	20	19	64.8±8.3	65.9±8.4	13/6	15/5
Maruko (2011)	Retro	1	Japan	6 m	11	16	71	71.8	6/5	12/4
Rouvas (2011)	Retro	2	Greece	12 m	9	11	64.67	62.9	4/5	5/6
Gomi (2010)	Retro	1	Japan	>12 m	61	85	70.9±7.1	70.9±6.8	45/16	68/17
Lai (2011)	Retro	1	Hong Kong	≥12 m	16	12	71.3±9.8	65.6±11.0	8/8	10/2
Sakurada (2013)	Retro	1	Japan	≥24 m	24	34	73.2±7.4	70.1±7.1	16/8	25/9
Kang (2013)	Retro	1	Korea	≥24 m	20	19	70.00±7.75	66.21±9.00	NA	NA

RCT  =  randomised controlled trials; Retro =  retrospective comparative studies; Combine  =  PDT plus intravitreal anti-VEGF inhibitors; PDT  =  photodynamic alone; M/F =  male/female; NA  =  not available.

**Table 2:** Characteristics of lesions and treatment exposures included in the meta-analysis.

Study	Group	Lesion GLD (µm)	Interventions	Number of treatments (mean±SD) (range)
Lee (2013)	Combine	NA	PDT+IVR 0.5 mg (<1 hour after PDT)	1PDT,1IVR
	PDT	NA	PDT	1PDT
Saito (2013)	Combine	4074±1459	IVR 0.5 mg+PDT (1 or 2 days after IVR)	1.4PDT, 4.5IVR
	PDT	4867±1855	PDT (6 mg/m^2^)	2.6PDT
Lee (2012)	Combine	2619±843	IVB 1.25 mg+ PDT (1 week after IVB)	2.25PDT, 2.42IVB
	PDT	2842±1092	PDT (6 mg/m^2^)	2.55PDT
Koh (2012)	Combine	<5400	PDT+IVR 0.5 mg (<24 hours after PDT)	1.4(1-4)PDT, 3.9(3-6)IVR
	PDT	<5400	PDT (6 mg/m^2^) + sham	1.7(1-4)PDT
Kim (2011)	Combine	3287.5 ±1335.9	PDT +IVB 1.25 mg (on the same day)	1.30±0.47 (1-2)PDT, 2.90±1.41(1-5)IVB
	PDT	3626.3±1334.6	PDT (6 mg/m^2^)	1.89±0.94 (1-4)PDT
Maruko (2011)	Combine	2905±1122	IVR 0.5 mg + PDT (1-2 day after IVR)	1PDT, 3IVR
	PDT	3013±1059	PDT (6 mg/m^2^)	1PDT
Rouvas (2011)	Combine	NA	IVR 0.5 mg+PDT (7±2 days after IVR)	1.67(1-2)PDT, 5(3-6)IVR
	PDT	NA	PDT (6 mg/m^2^)	1.82(1-3)PDT
Gomi (2010)	Combine	2626±1138	IVB 1.25 mg +PDT (1 day after IVB)	1.43PDT, 1.92IVB
	PDT	2521±996	PDT (6 mg/m^2^)	1.66PDT
Lai (2011)	Combine	3490±1170	PDT+ IVR 0.5 mg (30 min after IVR)	1.2(1-2)PDT, 3.4(3-6)IVR
	PDT	2580±707	PDT (6 mg/m^2^)	1.7(1-4)PDT
Sakurada (2013)	Combine	2039±847	IVR 0.5 mg+PDT (1 week after IVR)	1.54(1-3)PDT, 1.71(1-4)IVR
	PDT	2364±716	PDT (6 mg/m^2^)	1.42(1-3)PDT
Kang (2013)	Combine	2815±910.12	PDT +IVB 0.5 mg (on the same day)	1.67±0.65PDT,11.00±2.61IVB
	PDT	2810.87±974.10	PDT (6 mg/m^2^)	2.56±0.38 PDT

Combine  =  PDT plus anti-VEGF inhibitors; PDT  =  photodynamic alone; NA  =  not available; GLD  =  greatest linear dimension; SD  =  standard deviation; IVR  =  intravitreal ranibizumab; IVB  =  intravitreal bevacizumab.

### 2. Visual outcomes

VA was the most important criterion for evaluating efficacy. In the included studies, VA was measured using the logMAR scale and reported as the mean change in logMAR units from baseline for each group. In the combined PDT with anti-VEGF therapy group, the mean VA improved continuously during the two years of treatment when compared with baseline VA. The pooled WMDs at 3, 6, 12, and 24 months were 0.14 (0.07, 0.21), 0.17 (0.11, 0.24), 0.19 (0.12, 0.26), and 0.21 (0.11, 0.30), respectively. In the PDT monotherapy group, the mean VA improved at three and six months after initial treatment. However, it deteriorated after six months, and at 12 and 24 months, it was not significantly different from baseline VA.

**Figure 2:**
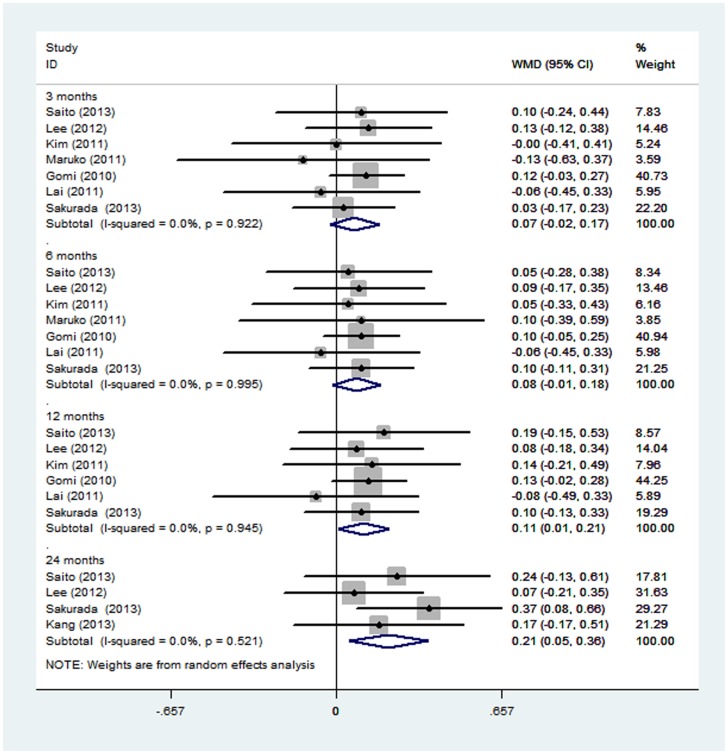
Forest plot displaying the pooled summary estimates of visual acuity in the combined PDT and anti-VEGF therapy group versus the PDT monotherapy group. VA = visual acuity.

LogMAR VA improvements in the combined PDT with anti-VEGF therapy group versus those in the PDT monotherapy group are shown in Figure 2 and Table 3. At three and six months post-injection, no significant difference in VA was found in the combined therapy group compared with the PDT monotherapy group, with pooled WMDs of 0.074 (−0.021, 0.17) at three months and 0.082 (−0.013, 0.18) at six months. However, the mean changes in BCVA at month 12 in the combined PDT with anti-VEGF therapy group were significantly better than those of the PDT monotherapy group, with pooled WMDs of 0.11 (0.012, 0.21). Similar efficacy was found at 24 months (WMD: 0.21; 95%CI: 0.054, 0.36; p = 0.008). There was no evidence of heterogeneity across trials.

**Table 3:** Results of meta-analysis comparison of combined therapy and PDT monotherapy.

Outcome of interest	Studies (n)	WMD/OR (95% CI)	P	Study heterogeneity		
				?2	P	I^2^
**Mean logMAR change in combined PDT and anti-VEGF therapy group (followup vs baseline)**						
3 months	7	0.14 (0.07, 0.21)	**<0.001**	4.84	0.565	0.00%
6 months	7	0.17 (0.11, 0.24)	**<0.001**	2.38	0.881	0.00%
12 months	6	0.19 (0.12, 0.26)	**<0.001**	4.92	0.426	0.00%
24 months	4	0.21 (0.11, 0.30)	**<0.001**	1.23	0.746	0.00%
**Mean logMAR change in PDT therapy group (followup vs baseline)**						
3 months	7	0.06 (−0.01, 0.12)	0.075	4.76	0.575	0.00%
6 months	7	0.09 (0.02, 0.15)	**0.010**	4.07	0.667	0.00%
12 months	6	0.06 (−0.01, 0.13)	0.075	4.93	0.424	0.00%
24 months	4	0.00 (−0.12, 0.12)	0.972	1.9	0.592	0.00%
**LogMAR improvements in both groups (combined PDT and anti-VEGF group vs PDT monotherapy group)**						
3 months	7	0.074 (−0.021, 0.17)	0.127	1.98	0.922	0.00%
6 months	7	0.082 (−0.013, 0.18)	0.092	0.67	0.995	0.00%
12 months	6	0.11 (0.012, 0.21)	**0.028**	1.2	0.945	0.00%
24 months	4	0.21 (0.054, 0.36)	**0.008**	2.26	0.521	0.00%
**LogMAR change as categorical variable**						
Proportion of eyes with improved vision						
final visit	6	1.91 (1.14, 3.18)	**0.013**	2.8	0.73	0.00%
12 months	3	2.33 (1.07, 5.07)	**0.033**	1.67	0.434	0.00%
24 months	3	1.64 (0.83, 3.22)	0.154	0.68	0.71	0.00%
Proportion of eyes with deteriorated vision						
final visit	8	0.77 (0.41, 1.46)	0.423	5.42	0.609	0.00%
12 months	5	0.87 (0.38, 1.98)	0.741	1.35	0.854	0.00%
24 months	3	0.42 (0.08, 2.29)	0.317	3.86	0.145	48.10%
Proportion of eyes with stable vision						
final visit	4	0.98 (0.66, 1.46)	0.926	1.41	0.702	0.00%
12 months	9	0.83 (0.47, 1.46)	0.512	5.52	0.700	0.00%
24 months	5	1.15 (0.67, 1.99)	0.617	3.44	0.487	0.00%
**Anatomical outcomes**						
CRT reduction at 6 months	4	26.19 (−15.38, 67.76)	0.217	1.81	0.614	0.00%
Resolution of PED at 12 months	4	2.18 (0.48, 9.89)	0.311	7.31	0.063	59.00%
Regression of polyps at 3 months	9	1.43(0.9, 2.27)	0.130	8.19	0.415	2.30%
Regression of polyps at 6 months	4	1.80 (0.66, 4.87)	0.248	2.07	0.557	0.00%
Recurrence rate of PCV	6	0.95 (0.59, 1.53)	0.840	1.87	0.867	0.00%
**Adverse events**						
Incidence of retinal haemorrhage	5	0.32 (0.14, 0.74)	**0.008**	4.22	0.378	5.10%

Combine  =  PDT plus intravitreal anti-VEGF inhibitors; PDT  =  photodynamic alone; NA  =  not available; logMAR  =  logarithm of the minimal angle of resolution; CRT  =  central retinal thickness; PED  =  pigment epithelial detachment; PCV  =  polypoidal choroidal vasculopathy; WMD  =  weighted mean difference; OR  =  odds ratio; CI  =  confidence interval; χ2  =  chi-square statistic; P  =  P-value; I^2^  =  I-square heterogeneity statistic.

When VA change was treated as a categorical variable, the percentages of improved, stable, and deteriorated VA were compared at 12 months, 24 months, and final visits. The rates of improved VA were significantly higher in the combined PDT with anti-VEGF therapy group than in the PDT monotherapy group at 12 months and final visits. However, the rates did not differ significantly at 24 months. Although the pooled results favoured the combined PDT with anti-VEGF therapy group in terms of stable and deteriorated VA, the differences were not statistically significant at any of the time points (Table 2).

### 3. Anatomical outcomes

CRT was defined as the distance between the internal limiting membrane and the inner surface of the retinal pigment epithelium, and measured at the fovea. CRT changes from baseline to month six were reported in four studies including 145 patients; the change in CRT was higher in the combined PDT with anti-VEGF therapy group than the PDT monotherapy group, but this difference was not statistically significant (WMD: 26.19; 95% CI: −15.38, 67.76; p = 0.217).

Four studies reported data for the frequency of PED resolved at 12 months. Analysis of these data showed no significant difference between the combined PDT with anti-VEGF and PDT monotherapy groups (OR: 2.18; 95% CI: 0.48, 9.89; p = 0.311).

Regression rates of polyps at three and six months were available for nine and four studies, respectively; there were no significant differences between the two groups (OR: 1.43; 95% CI: 0.90, 2.27; p = 0.130; and OR: 1.80; 95% CI: 0.66, 4.87; p = 0.248, respectively).

Recurrences of PCV during the follow-up periods were noted in six studies. Although the PDT monotherapy group showed more recurrences, there was no significant difference in recurrence rate between the groups (OR: 0.95; 95%CI: 0.59, 1.53; p = 0.840).

### 4. Adverse events

Retinal haemorrhage was the most common complication. Five studies including 340 patients reported frequency of retinal haemorrhage, and the pooled data showed a significant difference favouring the combined PDT with anti-VEGF therapy group (OR: 0.32; 95% CI: 0.14, 0.74; p = 0.008) (Table 2).

### 5. Subgroup analysis, sensitivity analysis and publication bias

There was no statistically significant difference in the mean changes of BCVA at 3 months and 6 months between combined PDT and anti-VEGF therapy group and PDT monotherapy group at all subgroups (Table 4). Visual outcome results were not significantly influenced by type of anti-VEGF agents, frequency of intravitreal anti-VEGF therapy, inteval between PDT and anti-VEGF therapy, and previous intevention history. Because of unadequate number of studies, subgroup analysis for other outcomes were not available. To evaluate the robustness of the results, each study in the meta-analysis was excluded in turn to reflect the influence of individual studies on the pooled estimates. The pattern of differences was similar to that of the original analysis, suggesting high stability of the meta-analysis results (data not shown). Begg's tests (all p>0.05) and Egger's tests (all p>0.05) showing no evidence of publication bias (data not shown).

**Table 4:** Subgroup analysis comparing combined PDT with anti-VEGF therapy group with PDT monotherapy group for change in LogMAR from baseline.

Subgroup	Studies(n)	WMD(95%CI)	P	Test for Heterogeneity		
				?2	P	I^2^
**LogMAR improvements at 3 months**						
All	7	0.07 (−0.02, 0.17)	0.127	1.98	0.922	0.00%
Ranibizumab used	4	0.02 (−0.14, 0.17)	0.838	0.73	0.867	0.00%
Bevacizumab used	4	0.11 (−0.01, 0.23)	0.072	0.31	0.856	0.00%
Protocol of anti-VEGF was 3+PRN	4	0.06 (−0.11, 0.23)	0.501	1.27	0.736	0.00%
Protocol of anti-VEGF was 1+PRN	3	0.08 (−0.03, 0.2)	0.165	0.66	0.720	0.00%
All patients were treatment-naïve	5	0.10 (−0.01, 0.21)	0.085	1.16	0.885	0.00%
Some patients receive intervention previously	2	0.01 (−0.17, 0.19)	0.904	0.16	0.687	0.00%
PDT 1-7 days after intravitreal anti-VEGF therapy	5	0.09 (−0.01, 0.19)	0.090	1.34	0.855	0.00%
PDT and anti-VEGF therapy on the same day	2	−0.03 (−0.32, 0.25)	0.826	0.04	0.836	0.00%
**LogMAR improvements at 6 months**						
All	7	0.08 (−0.01, 0.18)	0.092	0.67	0.995	0.00%
Ranibizumab used	4	0.07 (−0.09, 0.22)	0.400	0.53	0.911	0.00%
Bevacizumab used	3	0.09 (−0.03, 0.22)	0.138	0.06	0.972	0.00%
Protocol of anti-VEGF was 3+PRN	4	0.05 (−0.12, 0.22)	0.545	0.44	0.932	0.00%
Protocol of anti-VEGF was 1+PRN	3	0.10 (−0.02, 0.21)	0.104	0.06	0.971	0.00%
All patients were treatment-naïve	5	0.09 (−0.02, 0.2)	0.122	0.12	0.998	0.00%
Some patients receive intervention previously	2	0.07 (−0.12, 0.25)	0.486	0.51	0.477	0.00%
PDT 1-7 days after intravitreal anti-VEGF therapy	5	0.09 (−0.01, 0.2)	0.071	0.08	0.999	0.00%
PDT and anti-VEGF therapy on the same day	2	0 (−0.28, 0.27)	0.976	0.16	0.693	0.00%

PDT  =  photodynamic therapy; logMAR  =  logarithm of the minimal angle of resolution; 3+PRN =  3 initial monthly + as needed injection; 1+PRN = 1 initial monthly + as needed injection.

## Discussion

Among the currently available treatment modalities for PCV, PDT alone or PDT combined with VEGF inhibitors therapy seem the most promising [24], [25]. This meta-analysis of two RCTs and nine retrospective studies including 543 patients, comparing the efficacy of combined PDT with anti-VEGF therapy and PDT monotherapy, showed that combined PDT with anti-VEGF therapy was superior to PDT monotherapy in terms of visual outcome and complication. We found no significant differences in CRT reduction, polyp regression, PCV recurrence, or PED resolution.

The results showed that PDT alone will not be the best option to treat PCV, mainly until 6 months. PDT monotherapy causes regression of polyps and reduces fluid leakage, but polypoidal lesions have high recurrence rates over long periods. Furthermore, PDT is associated with the risk of submacular hemorrhages in PCV, which is confirmed in this study. The papers used in this meta-analysis did not explore the parameters of PDT and outcomes of treatment, which prevent our intution for disscussion. Anti-VEGF drugs reduced exudative fluid and suppressed upregulation of VEGF, but the vascular lesions did not regress. Thus, the combination of PDT and an anti-VEGF agent seems to be a rational approach.

In our study, the mean VA changes in the combined PDT with anti-VEGF therapy group were superior to those of the PDT monotherapy group at the 12-month and 24-month follow-up time points. Furthermore, the improvement in mean VA seemed to decrease with time in the PDT monotherapy group. In patients treated with PDT monotherapy, the improvement of mean VA did not reach statistical significance after six months. Theoretically, several additional PDT sessions would be necessary to treat PCV recurrences during longer follow-up periods [26]. It means an increase in the risks induced by PDT, such as subretinal haemorrhage and ischemic damage of normal choroidal tissue. PDT combined with an anti-VEGF drug can reduce those side effects of PDT [27]. Therefore, eyes in the combined therapy group had more potential to maintain better VA over the long term.

A similar rate of resolution of the original PCV vasculature was observed in the two treatments; the recurrence rates were also similar. This seemed reasonable, because various trials have shown that anti-VEGF agents are effective in reducing leakage, resolving fluids, and improving VA, but ineffective for polyp regression [7]–[9], [15], [28], [29]. Thus, the resolution of polypoidal lesions may be attributed mainly to PDT alone.

Retinal haemorrhage is one of the major problems associated with PCV treatment [8], [10]–[19]. In this study, we found a significantly lower rate of retinal haemorrhage in the combined PDT with anti-VEGF treatment group. It has been reported that PDT induces ischemia in the choroid, and that inflammatory responses around the RPE result in enhanced expression of VEGF [30]. Intravitreal injection of an anti-VEGF agent could therefore block the adverse effects induced by the increased VEGF expression, which might account for the lower retinal haemorrhage rate observed in the combined PDT group [31]. The reduced risk of haemorrhage induced by anti-VEGF inhibitors should encourage ophthalmologists to choose the combined therapy.

Although combined therapy showed better results than mono therapy, combined therapy was not sufficient to improve the results obtained mainly after 12 and 24 months of evaluation. Several studies have reported that the efficacy of PDT and PDT combined with intravitreal anti-VEGF agents decreased with time [11], [14], [24], [32]. Resolution of the original PCV vasculature was observed at a similar rate between the treatments, and the recurrence rates also were similar.Therefore, decrease in benefit at 12 months seems to be because of recurrences of exudative changes or atrophic tissue changes related to persistent lesions. In addition, the possible influence of cumulative harmful effect of PDT cannot be excluded. To maintain the beneficial effects of combination therapy with long-term follow-up, further investigation will be needed.

To our knowledge, this is the first meta-analysis comparing combined therapy and PDT monotherapy in the treatment of PCV. While heterogeneity is often a concern in a meta-analysis, little evidence of heterogeneity was observed throughout our study. This finding can be partially explained by the following facts: all of the studies used common indications and measurements of outcomes; all of the studies, except one [8], were conducted in Asian countries, where populations share much in terms of genetic background, lifestyle, and dietary patterns; and the baseline characteristics of most of the studies were comparable. To assess any impact of a single study on the effect estimates, we performed a sensitivity analysis by iteratively removing one study at a time to assess the stability of the meta-analysis results; the results were similar to those of the initial analysis. Although a meta-analysis of RCTs only would be ideal, the limited number of RCTs prevented us from reaching any definitive conclusions based on those studies alone.

The present meta-analysis has some limitations that must be taken into account. First, all the included studies were retrospective, except for two RCTs with small sample sizes. Inadequate random sequence generation and blinding could result in selection bias, as patients with worse visual prognoses might be offered the combination treatment. Nonetheless, the major characteristics of the eyes in the two groups were comparable at baseline, and therefore, selection bias was less likely to occur. Second, the combination group used either bevacizumab or ranibizumab as an anti-VEGF agent, and there might be a difference between the two agents when treating PCV. However, the results of the subgroup analysis showed that the effect of different anti-VEGF agents led to similar VA changes. Recent studies also have demonstrated that these two agents have similar efficacy in treating both age-related macular degeneration and PCV [33]–[36]. Third, because the studies included in the analysis were mostly conducted at major institutions, the patients evaluated might not reflect actual patient populations in the community. Fourth, while computer-based literature searching is essential, it is possible that not all of the relevant studies were identified, because ‘grey literature’ was not included in this study. Fifth, PDT combined with anti-inflammatory compounds and the combination therapy involving PDT, anti-VEGF and an anti-inflammatory agent did not evaluated. Further studies are necessary to evaluate these protocols for treating PCV. Finally, given that the treatment of PCV is not limited to two years, more data are needed from studies of longer duration in order to determine the efficacy and safety of combination therapy over the long term.

In conclusion, the present meta-analysis suggests that combination of PDT and anti-VEGF therapy results in better long-term visual outcomes and lower incidence rates of retinal haemorrhage than PDT monotherapy. The two treatments appear to be equivalent in terms of polyp regression and recurrence, CRT reduction, and PED resolution. Nevertheless, despite our rigorous methodology, the inherent limitations of the included studies should be considered, and conclusions drawn from our pooled results should be interpreted with caution. Future large-volume, well-designed RCTs with extensive follow-up are awaited to confirm and update the findings of this analysis.

## Supporting Information

Table S1 **The inclusion criteria of the patients in the studies included in the meta-analysis.**
(DOCX)

Checklist S1 **PRISMA checklist.**
(DOC)
