# Supplementary material for: Combined Intravitreal Anti-VEGF and Photodynamic Therapy versus Photodynamic Monotherapy for Polypoidal Choroidal Vasculopathy: A Systematic Review and Meta-Analysis of Comparative Studies
Source: PLoS One. 2014 Oct 24;9(10):e110667. doi: 10.1371/journal.pone.0110667 (PMC4208801; doi:10.1371/journal.pone.0110667)
Supplement: Table S1 — The inclusion criteria of the patients in the studies included in the meta-analysis. (DOCX) [file pone.0110667.s001.docx]

**Table S2.** The inclusion criteria of the patients in the studies included in the meta-analysis.

| **First author** | **Year** | **Inclusion criteria** |
| --- | --- | --- |
| Lee | 2013 | A diagnosis of PCV was established by the presence of polypoidal dilations, with or without a branching vascular network, on ICGA using a confocal scanning laser ophthalmoscope (Heidelberg Retina Angiograph; Heidelberg Engineering, Heidelberg, Germany). Patients with symptomatic PCV, considered as macular fluid on OCT and definite subfoveal, juxtafoveal, or extrafoveal macular polyps on ICGA, were included. |
| Saito | 2013 | All patients were treatment-naïve, and a clinical diagnosis of PCV was established based on the ICGA findings of polypoidal lesions. |
| Lee | 2012 | The diagnosis of PCV was made based on the finding of characteristic polyp-like hyperfluorescence with or without a branching vascular network on ICGA. The inclusion criteria were as follows: (1) juxtafoveal, subfoveal, or extrafoveal active macular polypoidal lesions on ICGA; (2) macula-involved active lesions, such as submacular fluid, recent hemorrhage, lipid exudates, and macular edema as evidenced by OCT or fluorescein angiography (FFA); (3) initial best-corrected visual acuity (BCVA) worse than 20/32 (0.2 logarithm of minimal angle of resolution [logMAR]); (4) greatest linear dimension (GLD) of the lesion of 5400μm or less; and (5) follow-up of more than 24 months. |
| Koh | 2012 | The key eligibility criteria were as follow: (1) BCVA letter score of 73 to 24 using Early Treatment of Diabetic Retinopathy Study charts at a starting distance of 4 m (～20/40 to 20/320 Snellen equivalent); (2) a GLD of the lesion of ,5400mm(～9 Macular Photocoagulation Study disk areas), assessed by ICGA; and (3) a confirmed diagnosis of PCV, that is, presence of early subretinal focal ICGA hyperfluorescence (appearing within the first 6 minutes after injection of indocyanine green) and in addition, at least one of the following angiographic or clinical criteria: (i) association with a BVN, (ii) presence of pulsatile polyp, (iii) nodular appearance when viewed stereoscopically, (iv) presence of hypofluorescent halo (infirst 6 minutes), (v) orange subretinal nodules in stereoscopic color fundus photograph (polyp corresponding to ICGA lesions), or (vi) association with massive submacular hemorrhage (defined as size of hemorrhage of at least 4 disk areas). |
| Kim | 2011 | The diagnosis of PCV was based on the presence of branching vascular networks and polypoidal choroidal vascular lesions on ICGA using scanning laser ophthalmoscope (Heidelberg Retina Angiograph; Heidelberg Engineering, Heidelberg, Germany). The inclusion criteria were as follows: symptomatic PCV in the macular area, age≥50 years, initial BCVA between 20/800 and 20/30, initial GLD≤6,000μm, and at least 12 months of follow-up period. |
| Maruko | 2011 | The diagnostic criteria for PCV were based on ICGA findings, which imaged the characteristic aneurysmal lesions. All patients with PCV had choroidal neovascularization on fluorescein angiography, which was identified as PCV on ICGA. Choroidal vascular hyperpermeability, seen as hyperfluorescence in middle-phase ICGA images, also was evaluated |
| Rouvas | 2011 | Inclusion criteria were (1) macular or temporal peripapillary PCV with baseline visual acuity (VA) of 20/30 or worse; (2) identification of polyps and interconnecting vessels on the ICGA (dilated network of vessels with multiple terminal aneurysmal protuberances in a polypoidal configuration ), located in the macula within 1 disk diameter from the center of the foveal avascular zone; and (3) presence of subretinal hemorrhages and/or exudation in the macula based on clinical examination. |
| Gomi | 2010 | The diagnosis of PCV was established based on the finding of polyplike choroidal vessel dilatation with or without a branching vascular network on ICGA. Eyes with either polypoidal lesions or regions of branching vascular networks in the subfovea were included. |
| Lai | 2011 | The inclusion criteria were (1) age≥18 years; (2) PCV as defined by the presence of branching network of choroidal vessels with terminating aneurysmal polypoidal lesions in ICGA ; (3) logarithm of minimal angle of resolution (logMAR) BCVA from 0.1 to 1.6 or better (Snellen equivalent of 20/25–20/800); and (4) follow-up of ≥12 months |
| Sakurada | 2013 | Consecutive PCV patients with a baseline BCVA of 0.1 or better in the decimal scale, treatment naive and were followed up for 24 months or longer. |
| Kang | 2013 | The inclusion criteria were: (1) symptomatic treatment-naïve macular PCV with subfoveal leakage on FFA; (2) presence of branching vascular networks and polypoidal lesions on ICGA; and (3) follow-up at least 2 years after initial treatment. |
